# Components and Mechanisms: How Children Talk About Machines in Museum Exhibits

**DOI:** 10.3389/fpsyg.2021.636601

**Published:** 2021-05-28

**Authors:** Elizabeth Attisano, Shaylene E. Nancekivell, Stephanie Denison

**Affiliations:** ^1^Department of Psychology, University of Waterloo, Waterloo, ON, Canada; ^2^Department of Psychology, University of North Carolina at Greensboro, Greensboro, NC, United States

**Keywords:** informal learning, cognitive development, machines, mechanisms, museums

## Abstract

The current investigation examines children’s (*N* = 61; 4- to 8-year old) learning about a novel machine in a local history museum. Parent–child dyads were audio-recorded as they navigated an exhibit that contained a novel artifact: a coffee grinder from the turn of the 20th century. Prior to entering the exhibit, children were randomly assigned to receive an experimental “component” prompt that focused their attention on the machine’s internal mechanisms or a control “history” prompt. First, we audio-recorded children and their caregivers while they freely explored the exhibit, and then, we measured children’s learning by asking them two questions in a test phase. Children of all ages, regardless of the prompt given, discussed most aspects of the machine, including the whole machine, its parts, and, to a lesser extent, its mechanisms. In the test phase, older children recalled more information than younger children about all aspects of the machine and appeared more knowledgeable to adult coders. Overall, this suggests that children of all ages were motivated to discuss all aspects of a machine, but some scaffolding may be necessary to help the youngest children take full advantage of these learning opportunities. While the prompts did not significantly influence the number of children who discussed the machine’s mechanisms, children who received the component prompt were rated as more knowledgeable about the machine in the test phase, suggesting that this prompt influenced what they learned. Implications for visitor experience and exhibit design are discussed.

## Introduction

When encountering a new artifact, children have much to learn, including facts relevant to the whole artifact, such as its name and purpose, and facts about its components such as the role of specific parts in its operation. Mechanical machines provide a particularly unique learning challenge for young children, as they consist of not only external parts but also internal parts and mechanisms that are unseen but critical to their functioning (e.g., Leuchter and Naber, 2018; Reuter and Leuchter, 2020). Reflecting this fact, early childhood science curricula emphasize the importance of teaching young children about mechanical machines and forces during grade school (e.g., Ontario Ministry of Education, 2007; Michigan Department of Education, 2015). For developmental scientists, mechanical machines provide an opportunity to explore children’s causal reasoning (e.g., Legare et al., 2010; Sobel et al., 2020). The present investigation seeks to understand how children learn about mechanical machines during interactions with their caregivers in more informal, naturalistic contexts than those of schools or laboratories.

Our main questions are what information do children discuss when learning about novel mechanical artifacts in museum exhibits and how might short verbal instructions or prompts influence children’s discussions and learning? To do this, we examined how children talk and learn about a novel artifact – a coffee grinder (circa 1914) found in a local social history museum. We provided children with one of two verbal prompts directing their attention to the internal mechanisms of the machine (experimental prompt) or a neutral control prompt. We focus on an informal learning environment because the minimal educational structure can reveal how learning about such artifacts unfolds when primarily driven by unstructured exploration (e.g., Sobel and Jipson, 2016). This unstructured exploration in a living history exhibit, which is not specifically geared toward learning about novel causal mechanisms, may provide insight into how children acquire these concepts in the course of their everyday lives. It also provides information for educators and designers in these spaces who hope to promote particularly rich and varied learning opportunities for children.

Our first aim was to document how children talk about mechanical machines in museums when visiting with their families. When examining a novel machine, a child might choose to focus on the whole machine (such as its name, what it is made out of, and its function and purpose), the machine’s parts (both external and internal), and the mechanism of its operation. All of these aspects are important for understanding the machine’s operation. Previous work has documented that children are particularly adept at learning about an artifact’s function and purpose (Casler and Kelemen, 2005, 2007). Children also expect people to use artifacts in a normative way, as opposed to in atypical ways (Casler et al., 2009; Weatherhead and Nancekivell, 2018). From a young age, children view the function and purpose of an artifact as important features to learn (Kemler Nelson et al., 2004; Greif et al., 2006), along with the artifact’s identity (Kelemen, 1999; Matan and Carey, 2001; German and Johnson, 2002). Additionally, children as young as 3 years old in laboratory tasks acknowledge that the insides of an artifact are important to its function and identity (Gelman and Wellman, 1991).

A great deal of work in cognitive development has focused on children’s reasoning about, and attention to, artifacts’ internal mechanisms (e.g., Sobel et al., 2007; Ahl and Keil, 2017; Ahl et al., 2020). For example, 4-year-old understands that an object’s internal component can activate a machine, and they expect other objects with the same internal component to work in similar ways (Sobel et al., 2007, see also Walker et al., 2014). Children are also able to reason about the diversity of a machine’s functions and how this relates to the complexity of a machine’s insides (Ahl and Keil, 2017, see also Erb et al., 2013 for related findings). Further to this, children understand that complex objects require expert knowledge to be used or fixed (Kominsky et al., 2018). Some research has also focused on children’s understanding of the internal mechanisms of machines in museum settings. This work shows that parents play a vital role in directing children’s attention to important features of machines (e.g., Callanan et al., 2020; Medina and Sobel, 2020; Pagano et al., 2020). For example, children will discover more properties and gain a deeper understanding of the underlying mechanisms and internal components of a machine when they explore with their parent, rather than on their own or with a peer (Crowley et al., 2001; Fender and Crowley, 2007).

Together, this work highlights the importance of examining children’s understanding of machines and their components, as they relate to causal reasoning and STEM education. Because the machines at the museum in this article are from the early 20th century, they are novel and involve only manual parts and mechanisms, allowing children to identify the problem these machines solve and hypothesize about how their parts and internal components aid in its operation, all of which children have been shown to have an appreciation for laboratory settings (e.g., Casler and Kelemen, 2005; Ahl and Keil, 2017). This practice provides foundational knowledge for understanding the more complex machines and technology found in the 21st century.

Our second aim was to understand how providing a minimal verbal prompt to children might affect their discussions with their parents about a machine in a museum exhibit. Prior work has established that children are more engaged when adults provide explanations (Frazier et al., 2009) and produce more on-topic utterances when their parent asks them causal questions (Benjamin et al., 2010; Rowe et al., 2017; Chandler-Campbell et al., 2020). As such, prior work has focused on how providing parents and children with supplementary materials and prompts can enhance their learning in exhibits (e.g., Benjamin et al., 2010; Haden et al., 2014; Callanan et al., 2017; Chandler-Campbell et al., 2020; Pagano et al., 2020). Most of this work employs conversational cue cards to parents to encourage them to interact with and explain information to their child. For example, in an African history exhibit, giving families materials suggesting what to look for in the exhibit (i.e., written prompts) and prompts related to the exhibit influenced the amount of time spent at the exhibit (Tenenbaum et al., 2010). Similarly, a prompt on a cue card encouraging parents to promote explanations in their children leads children to spend more time testing the causal mechanisms of the gears in a gear exhibit, whereas a prompt to encourage exploration leads children to spend more time building complex gear machines (Willard et al., 2019). This suggests that prompting explanations leads to a greater causal understanding of how a machine operates, whereas a prompt to explore leads to increased engagement in the exhibit. Moreover, the presence of physical objects that parent–child dyads are able to manipulate also impacts how they engage with exhibits in a natural history museum (Jant et al., 2014; also see findings about “conversation cards”).

These studies show that directing interventions at both parents and children influences how children engage in exhibits. At the same time, minimal verbal prompts directed specifically at children in laboratory settings have successfully guided their learning toward causal properties of artifacts. For example, asking a child to explain why a block did not activate a machine, rather than recall if the block activated the machine, led children to privilege causal properties over perceptual similarity when making novel inferences (Walker et al., 2014). Therefore, we aimed to connect these findings from laboratory settings to informal learning environments by examining whether prompts directed only at children in informal settings will also influence their learning.

### The Present Study

Building on this work, we examined children’s learning about a novel artifact in a living history museum. We had children explore the exhibit with parents present, because this is how children would typically engage in this museum and because previous literature suggests that the presence of parents is beneficial to children’s learning in museums (Crowley et al., 2001; Fender and Crowley, 2007). The study began with a prompt phase, where we provided only children with one of two minimal verbal prompts (experimental or control). While previous studies have provided prompts to parents and children (e.g., Benjamin et al., 2010; Haden et al., 2014), we were interested in examining whether providing a prompt directly and exclusively to the children would influence their talk and learning for two reasons: First, this ensures that any effect of the prompt is driven by children, deconfounding this from contributions that might come from the parent. Second, this also benefits our partner museum, as children visit the museum with varying degrees of adult support, sometimes attending with their families or friends and sometimes on school trips. Following the prompt phase, children explored the artifact (learning phase) with their parents and with museum staff present, with audio recorded. Finally, in a test phase, children were asked two open-ended questions: one that probed all information they gained about the artifact and another that probed an explanation of how the artifact worked.

### The Setting

We undertook this investigation in a local social history museum and specifically examined how children learn about a coffee grinder in use in 1914. Most research examining children’s learning in informal environments occurs in highly interactive children’s museums or science exhibits explicitly aimed to engage and teach children about science concepts (e.g., Sobel and Jipson, 2016). In contrast, the historical museum we targeted promotes visitor-driven learning and exploration for people of all ages, not directly aimed at science learning. The museum where the experiment took place contains an indoor exhibit that describes the history of the Waterloo Region, as well as a 60 acre living history exhibit that aims to teach children and their families about local social, economic, and technological history by transporting visitors to the year 1914. This particular setting is a middle ground between a museum exhibit and the real world, as it contains hundreds of novel artifacts to discover and learn about, but it also resembles everyday life where children encounter scientific concepts. In a historical museum, a recent interest of staff and management is to identify the wide variety of learning opportunities to children, including those relating to scientific concepts.

The museum is located in a suburban area of a midsized Canadian city (Kitchener-Waterloo, Ontario). Admission is $11 CAD for adults and $5 CAD for children aged 5- to 12-year old, with free parking. Passes to visit the museum for free are also made available through local city libraries. The exhibits in the living history village are buildings that immerse visitors in 1914. Here, learning is mainly driven by the visitor themselves including their ability to ask questions, read accompanying guidebooks, and/or physically explore the space. There is little to no educational signage or direction provided to visitors, except for strategically positioned staff members,^1^ to maintain the illusion to visitors that they have been transported to the year 1914. As such, our goal of testing the impact of *verbal prompts* was particularly useful for our partner museum and any other museums with similar constraints.

## Materials and Methods

### Participants

All participants were recruited from Southwestern Ontario *via* onsite recruitment, social media advertisements, and a university database. All experiments were conducted with written informed consent obtained from a parent or guardian for each child before any assessment or data collection. All procedures involving human subjects in this study were approved by the University of Waterloo Research Ethics Board. Participants include 61 parent–child dyads. Children were between the ages of 4- and 8-year old, randomly assigned to two conditions: a component prompt and a control prompt. Demographic information was completed on behalf of children by their accompanying parent or guardian. In the final sample, 45 participants were identified as White, 33 participants reported an annual household income of over $100,000 CND, and 39 participants reported that the primary caregiver attended a 4-year university or held an advanced/professional designation. Please see https://osf.io/dxg7h/ for full participant demographic information.

Participants were tested between June and August 2019, as this encompasses a single season in the museum, which only operates in summer months. Thus, we aimed to test as many children as possible over this period, with the expectation of testing at least 30 children per condition. Prior work employing similar open-ended investigations in museums suggests that this sample size was adequate for investigating the present questions (e.g., Benjamin et al., 2010; Chandler-Campbell et al., 2020). As a thank you for participating, participants were given a family pass to come back to the museum, valued at $25 CAD. Fifteen additional dyads were tested but not included in the analyses for the following reasons: parental reported developmental disorder (8), parents answering test questions for their child (3), and child noncompliance (4; e.g., indicating they did not wish to participate anymore). Some participants had siblings present when they arrived to complete the study; if this was the case, siblings stayed away from the exhibit.

### Materials and Procedures

Participants were greeted by the experimenter upon entering the museum, where written informed consent was acquired. Therefore, participants did not enter the exhibit that day before the experiment took place.

**Figure 1:**
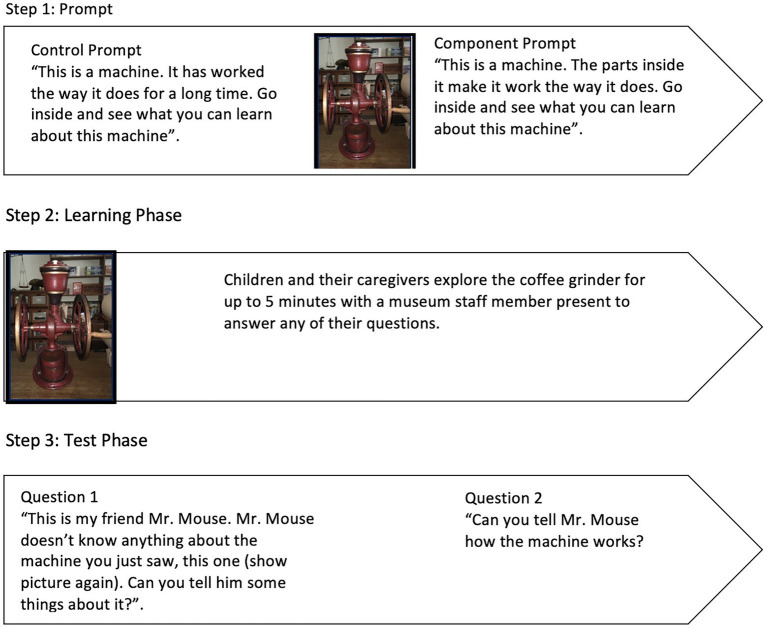
Visual schematic of the procedure.

Participants were led to the general store, where the machine (i.e., coffee grinder) was located. All interactions were audio-recorded using a Zoom Q2n-4k camera fitted to the child’s chest using a GoPro Junior Chesty with the camera lens blocked. The experiment was broken into three phases; the prompt phase, the learning phase, and the test phase (see Figure 1 for a schematic of the procedure).

#### The Machine

**Figure 2:**
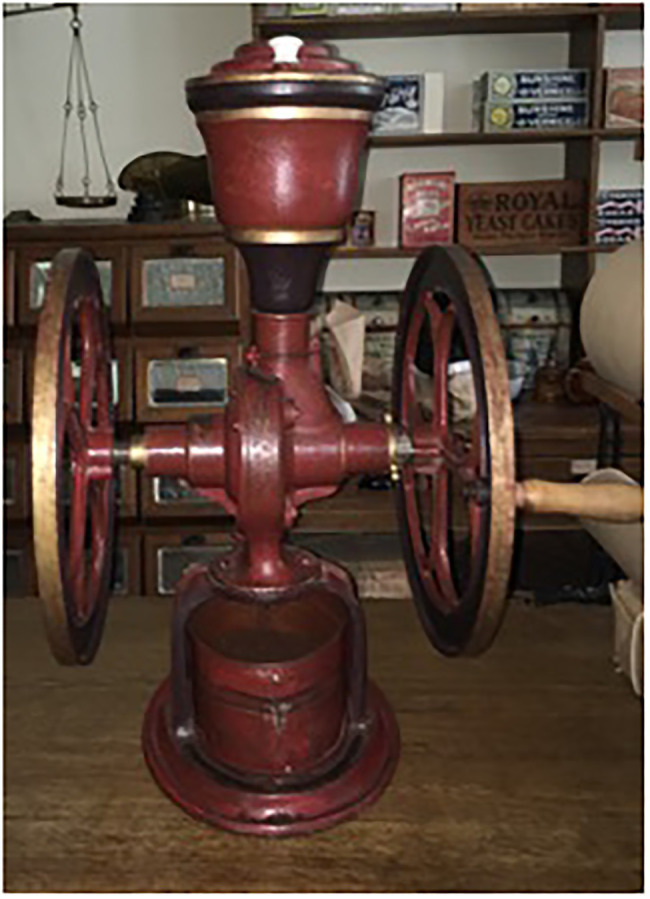
Coffee grinder used in this experiment.

The machine was a coffee grinder in use in 1914 (see Figure 2 as well as the supplement for an expert explanation of the coffee grinder’s operation). This machine was made of cast iron with two large wheels on either side. The top of the machine contained a tin with a lid, where one puts the coffee beans into the machine. The beans would then fall deeper in the machine to the grinders. One would need to turn the two large wheels on the side to activate the machine and grind the coffee beans. The grinds would fall out of the machine and get collected in a bin.

#### Prompt Phase

Prior to the learning phase, outside of the general store where the coffee grinder was located, children were briefly separated from their parent and given one of two prompts. Thirty children (12 males, *M*_age_ = 6.634 years, *SD* = 1.463)^2^ received the experimental component prompt “This is a machine. The parts inside of it make it work the way it does. Go inside and see what you can learn about this machine.” This prompt was designed to focus children on the machine’s mechanisms, while avoiding the jargon “mechanism,” which young children may not know. Previous experimental paradigms reveal that both adults and children as young as 5-year old rated characters who provide mechanistic explanations about mechanical machines as more knowledgeable than those that provide non-mechanistic explanations (Lockhart et al., 2019) and believe this mechanistic knowledge should be generalizable to related machines (Chuey et al., 2020). This suggests that children privilege mechanistic explanations, therefore prompting children to focus on mechanisms, will increase their talk about mechanisms and lead them to recall more mechanistic information at test.

Thirty-one dyads (13 males, *M*_age_ = 6.089 years, *SD* = 1.058) received a control prompt “This is a machine. It has worked the way it does for a long time. Go inside and see what you can learn about this machine.” This neutral control prompt was designed to be as equivalent as possible to the component prompt. That is, it still references a machine “working” but is otherwise neutral against the historical backdrop of the immersive museum experience and does not reference the critical “parts inside” (i.e., the mechanisms).

#### Learning Phase

After children received the prompt, parents and children entered the store to explore the machine. While we only measured and reported the verbal discussions of children and their parents, they were free to explore the machine in any way they wanted, which included touching the coffee grinder and moving it physically, although this was not captured due to recording audio only. Parents were told “You and your children will explore the coffee grinder at the Dry Goods and Grocery Store. You can talk about any aspect of the coffee grinder; feel free to interact with your child as you normally would. You can talk about the coffee grinder as long as you would like, I’ll come get you when time is up.” The experimenter was on the opposite side of the store, turned away from the participants, and appeared to be sorting through paperwork. Museum staff was present to answer questions from the parents or children. Beforehand, museum staff was instructed to interact with participants as they normally would: to provide information when requested and to otherwise let them discuss the machine themselves. Dyads were given a maximum of 5 min to discuss about the machine. At the 5-min mark or when the dyad indicated they were done investigating, the experimenter would begin the test phase.

#### Test Phase

After the learning phase, experimenters took the child either to the other side of the store or outside the store, depending on weather and the number of visitors in the space to complete the test phase. In the test phase, children were asked two test questions to assess how much and what they had learned. Parents were nearby and were instructed by the experimenter to not assist their child in answering the questions. To ensure that children’s beliefs about the experimenter’s prior knowledge did not influence the findings, the questions were asked on behalf of “Mr. Mouse” (a puppet), a naive learner. The first question was included to assess what children had learned about the machine and to extract as much information from each child as possible: “This is my friend Mr. Mouse. Mr. Mouse does not know anything about the machine you just saw, this one (show picture of the coffee grinder). Can you tell him some things about it?” The experimenter continued to prompt the child, using the interview probing technique, “Can you tell him something else?” until the child indicated they had nothing more to say.

The second question was designed to more directly target children’s ability to explain how the machine worked in a succinct explanation and thus targeted what children believed was *causally important* for the machine’s operation (as opposed to the quantity of what they knew as in question 1): “Can you tell Mr. Mouse how the machine works?” For this question, children were not repeatedly prompted as in question 1.

### Transcription and Coding

Each participant’s audio recording was transcribed and then broken into utterances by a research assistant. An utterance was operationalized as a continuous unit of speech without pauses, interruptions, or changes in subject (e.g., typically an independent clause). A second research assistant reviewed the transcripts for errors. The transcripts were found to be accurate by the second research assistant. This process resulted in the identification of 1,627 utterances spoken by children.

To prevent bias, age, gender, and condition of the child and identity of the parent were removed from transcripts before coding. The primary coder was unaware of the hypotheses of the study, whereas the first author was the secondary coder. Prior to coding, the primary and secondary coders coded five of the excluded participants for training purposes. The test phase was also coded separately from the learning phase (i.e., on a different day), at which time the coder could not see any data from the learning phase. The secondary coder reliability coded 30% of the participants.

#### Learning Phase Coding

The following coding was done for child speakers.

##### Total Talk

As a first step, a research assistant identified utterances that were related to the coffee grinder. This was done to filter out talk not directly related to the artifact of interest (e.g., talk about the store and other artifacts present). Through this process, 1,233 child utterances were identified as pertaining to the machine. Reliability was excellent with a kappa of 0.987 (Landis and Koch, 1977). A subset of this talk (507 utterances) consisted of content-free responses to adults, such as “yes” or “mhmm.” Although this was technically related to the artifact due to the context provided by the caregiver or staff person, these were not coded into the schemes that follow. Therefore, a total of 726 utterances are used in the following analyses.

##### Talk About the Whole Machine vs. Talk About Its Parts/Components

Utterances referring to the whole object included what a coffee grinder is, its name, history, its appearance and/or what it was made of (“It’s way older,” “It’s made of metal and steel”). Utterances referring to parts or components of the coffee grinder included its handle, gears, and wheels (“You spin this handle here,” “The stuff goes in the top here”). Reliability was excellent with a kappa of 0.962 (Landis and Koch, 1977).

##### Mechanistic Talk

The third scheme aimed to capture talk about the components or mechanisms that underlie the operation of the coffee grinder. For an utterance to be defined as mechanistic, it must identify a component of the coffee grinder and explain how or why that particular component operates the way it does (“So you turn, what you see when I’m turning right here. Then it grinds the coffee, the gears inside of it,” Lockhart et al., 2019). From this, speakers were given a score of 0 (indicating there were no mechanistic utterances) or 1 (indicating there was at least 1 mechanistic utterance). We used this binary coding because very few speakers made mechanistic utterances (18 participants), and those that did tended to make multiple such utterances. To prevent a small number of participants from skewing the data, we used binary coding rather than counts. Reliability was excellent with a kappa of 1 (Landis and Koch, 1977).

#### Learning Phase Hypotheses

This coding allowed us to explore which aspects of the machine children were most drawn to discussing, how children’s discussions evolve with age, and how the prompts influenced them. In terms of our prompts, we predicted that children who received the components prompt would have their attention drawn to the mechanisms of the coffee grinder. This might also result in them producing more utterances about the parts of the machine than children who received the history (control) prompt. The whole talk variable was included to examine how much children this age talk about the whole artifact, with no specific predictions about how the prompts might affect this talk, given that neither prompt was specifically designed to influence whole talk. Thus, this variable was included to examine whether the components or history prompt might have inadvertently influenced another variable (i.e., it is important to ensure that the experimental prompt did not inflate all types of relevant talk or that the control prompt did not somehow inflate whole object talk, pulling focus away from the mechanisms and internal part talk). Additionally, we anticipate effects of age, with older children having more discussions about the parts of the machine, and more mechanistic utterances, as this is in line with previously documented gains in education research (Reuter and Leuchter, 2020).

#### Test Phase Coding

Children’s answers to the two test questions were coded on different days by the primary coder to prevent one set of codes from influencing another.

Question 1 of the test phase, which asked children to recall facts about the machine [“Can you tell him (Mr. Mouse) some things about it?”], was coded similarly to the learning phase, with some notable exceptions: Total talk was not included, as all child utterances should be related to the coffee grinder. Reliability was excellent with a kappa of 0.965 for whole and part talk and excellent with a kappa of 0.948 for mechanistic talk (Landis and Koch, 1977).

Question 2 of the test phase, which asked children to explain how the machine worked (“Can you tell Mr. Mouse how the machine works?”), was coded using the same coding as question 1,^3^ as well as a global knowledgeability rating of the produced explanation. This knowledgeability rating aimed to capture the quality of children’s explanations by having two coders, naïve to study hypotheses, and rate on a 0–5 scale how knowledgeable the child was about the workings of the machine. As it was a judgment rating, the primary coder and another coder who was also unaware of the hypotheses of the study coded 100% of the participants. Both coders were given explanations as to how the coffee grinder operated by the first author (see Supplementary File). Coders gave the child a score from 0 to 5, with 0 indicating that the child did not answer the question, 1 indicating that the child did not know much about the coffee grinder, and 5 indicating that the child knew almost everything (see Supplementary File for examples). As the coders ratings were highly correlated (*r* = 0.870, *p* < 0.001), an average of the two scores was used for subsequent analyses.

#### Test Phase Hypotheses

This coding scheme allowed us to test which facts about the machine children learned, how their learning evolves with age, and how their learning was influenced by our prompts. We predicted that children who heard the component prompt would recall more facts about the parts and mechanisms of the machine in both questions compared to children who heard the control prompt. We also predicted that these children would be rated as more knowledgeable in question 2 than those that received the control prompt. Coders did not rate knowledgeability for question 1, because the key aim of the knowledgeability rating was to determine whether children became more knowledgeable specifically about the workings of the machine, and question 1 prompted children to divulge all aspects of the information they gained. We predicted that children who received the component prompt would be rated as more knowledgeable because prior work shows that explanations that reference the internal mechanisms and parts of a machine tend to appear more knowledgeable than those that provide non-mechanistic explanations (Lockhart et al., 2019; Chuey et al., 2020). Again, we also predicted the effects of age, with older children recalling more about the machine’s parts and mechanisms (Reuter and Leuchter, 2020).

## Results

All data and supplementary information can be found at: https://osf.io/dxg7h/.

### Learning Phase

When learning about the machine, children discussed most aspects of the machine, producing 11.902 relevant utterances (*SD* = 8.833) on average. In terms of talk about the whole machine, children discussed what it was made of, where it was made, and how old it is (*M* = 4.246 utterances, *SD* = 3.585). When learning about its parts, children discussed the opening where you add coffee beans, the bin where you collect the grinds, and its wheel (*M* = 4.738, *SD* = 4.423). Mechanistic utterances included identified a component of the coffee grinder and explained how or why that particular component operates the way it does (*M* = 0.295, *SD* = 0.459).

We ran a series of generalized linear models (GLMs) to test our hypotheses. For all analyses, frequency of target talk (i.e., total, part, whole, and mechanistic) was the dependent variable, condition (component vs. control prompt) was entered as a between subjects factor, and age in months entered as a mean-centered covariate, to control for any effects of age on the other variables of interest. Here and in the test phase, the total amounts of talk, amounts of whole object talk, and amounts of talk about object components were analyzed using a quasi-Poisson-based model. We planned to use a Poisson-based model, but there was significant over-dispersion for all of these dependent variables (they violated the Poisson model’s assumption of mean = variance), making quasi-Poisson-based models a better and more conservative choice. Children’s mechanistic scores (coded as 0/1) were analyzed using a binary logistic model.

For the GLMs for each dependent variable, there were no main effects of condition, no main effects of age, and no interactions for any of the dependent variables, except for a main effect of age for total talk^4^ (*t* = 2.862, *p* = 0.006) and whole talk (*t* = 2.900, *p* = 0.005; see Table 1 for all statistical tests).

**Table 1:** Learning phase statistical tests and means.

	Statistical test			Control prompt mean (SD; range)	Component prompt mean (SD; range)	Total mean (SD)
		*t*	*p*			
**Total**						
	Age	2.862	0.006^**^	10.548	13.3	11.902
	Condition	−0.448	0.656	(6.908)	(10.396)	(8.833)
	Condition × age	−0.307	0.760	(0–27)	(1–45)	
**Whole**						
	Age	2.900	0.005^**^	4.226	4.267	4.246
	Condition	0.901	0.371	(3.253)	(3.956)	(3.585)
	Condition × age	0.764	0.448	(0–15)	(0–17)	
**Part**						
	Age	1.732	0.089	3.903	5.600	4.738
	Condition	−1.08	0.285	(3.986)	(4.746)	(4.423)
	Condition × age	0.311	0.757	(0–14)	(0–22)	
**Mechanistic**						
	Age	0.353	0.553	0.258	0.333	0.295
	Condition	0.202	0.653	(0.445)	(0.479)	(0.459)
	Condition × age	0.168	0.682			

Mechanistic data are binary and are analyzed using a binary logistic model generalized linear model (GLM). Therefore, it is reported with a WaldX^2^.

** *p* < 0.01.

One potential concern is that the control prompt might have focused children’s attention to historical information about the machine or about the setting more broadly, taking focus away from mechanisms in that condition. Thus, historical utterances were coded for both children and parents/staff in the learning phase (see supplement for parental analyses).^5^ The coder was instructed to code any references to how old the machine was, using phrases such as “a long time ago,” “back in the olden days,” “1914,” or comparisons between old vs. new, then vs. now. For children, when analyzed using a quasi-Poisson GLM (*M* = 0.361, *SD* = 1.081), we found no main effect of age (*t* = 0.497, *p* = 0.621), condition (*t* = 0.503, *p* = 0.617) or condition by age interaction (*t* = 1.001, *p* = 0.321). Therefore, the control prompt did not lead children to discuss the more historical aspects of the machine at higher rates.

#### Learning Phase Correlations

Next, we examined how parent and museum staff engagement was related to children’s engagement. We coded parent and staff utterances using the same coding scheme as with children. The number of children who discussed about the machine in general (*r* = 0.304, *p* < 0.001), the whole machine (*r* = 0.546, *p* < 0.001), and its components (*r* = 0.460, *p* < 0.001) was correlated with the parent and museum staff discussions of each respective type of talk. Children’s mechanistic score was not related to the parent and museum staff’s mechanistic score (*r* = 0.153, *p* = 0.239).

### Test Phase

The second aim of the investigation was to determine whether the verbal prompts differentially influenced children’s learning about machines.

For test question 1, all types of talk increased with age (see Table 2). When children recalled facts about the whole machine, they recalled what it was called and how old it was (“It’s a hundred and 5 years old,” *M* = 1.213, *SD* = 1.462). When recalling the facts about the machine’s parts, they recalled the handles and wheels of the machine (“It grinds more coffee every time you roll the wheels,” *M* = 2.819, *SD* = 2.306). Mechanistic utterances included discussions about mechanisms (“You spin the wheel and it grinds the beans,” 22 participants, *M* = 0.361, *SD* = 0.484). There was no main effect of condition and no interaction (see Table 2).

**Table 2:** Test phase statistical tests and means.

	Statistical test			Control prompt mean (SD; range)	Component prompt mean (SD; range)	Total mean (SD)
		*t*	*p*			
**Question 1 whole**						
	Age	2.726	0.008^**^	1.193	1.233	1.213
	Condition	0.295	0.769	(1.492)	(1.455)	(1.462)
	Condition × Age	0.810	0.421	(0–6)	(0–5)	
**Question 1 part**						
	Age	2.403	0.019^*^	2.548	3.100	2.819
	Condition	−0.700	0.486	(2.488)	(2.107)	(2.306)
	Condition × age	1.620	0.111	(0–8)	(0–7)	
**Question 1 mechanistic**						
	Age	4.588	0.032^*^	0.355	0.367	0.361
	Condition	0.143	0.706	(0.486)	(0.490)	(0.484)
	Condition × age	0.351	0.554			
**Question 2 part**						
	Age	4.532	<0.0001^**^	1.710	2.267	1.984
	Condition	−0.547	0.587	(1.553)	(1.437)	(1.512)
	Condition × age	0.676	0.502	(0–6)	(0–5)	
**Question 2 mechanistic**						
	Age	7.678	0.006^**^	0.194	0.333	0.262
	Condition	0.297	0.586	(0.402)	(0.479)	(0.443)
	Condition × age	0.207	0.649			
**Knowledge**						
	Age	24.935	<0.001^**^	2.129	2.967	2.541
	Condition	4.902	0.027^*^	(0.991)	(1.332)	(1.236)
	Condition × age	0.043	0.836	(0–3.5)	(0–5)	

Mechanistic data are binary and are analyzed using a binary logistic model GLM, while knowledge ratings are analyzed using a linear model GLM. Therefore, both are reported with a WaldX^2^.

* *p* < 0.05;

** *p* < 0.01.

For test question 2, both part (*M* = 1.984, *SD* = 1.512) and mechanistic (16 participants, *M* = 0.262, *SD* = 0.443) talk increased with age (see Table 2). There were no whole talk utterances for any participant for this question. This is unsurprising, as children were directed to explain how the machine operated.

Knowledge ratings were analyzed using a linear model with the average ratings (0–5) as the dependent variable. There was a main effect of age [*WaldX^2^* (*df* = 1) = 24.935, *p* < 0.001] and a main effect of condition: children who received the component prompt (*M* = 2.967, *SD* = 1.332) were rated as more knowledgeable than children who received the control prompt [*M* = 2.129, *SD* = 0.991; *WaldX^2^* (*df* = 1) = 4.902, *p* = 0.027]. There was no condition by age interaction *WaldX^2^* (*df* = 1) = 0.043, *p* = 0.836.

Next, we examined how children’s talk in test question 1 related to their knowledge rating in test question 2. Whole talk was not significantly correlated with children’s knowledge rating (*p* = 0.080). However, both part talk (*r* = 0.383, *p* = 0.002) and mechanistic scores (*r* = 0.267, *p* = 0.037) were significantly correlated with children’s knowledge ratings. Children who recalled more facts about parts and mechanisms when asked about the machine more globally are likely to produce an explanation in the next phase that seems to convey high knowledgeability. Additionally, we examined how children’s talk in test question 2 related to their knowledge rating in question 2. Both part talk (*r* = 0.665, *p* < 0.001) and mechanistic scores (*r* = 0.649, *p* < 0.001) were significantly correlated with children’s knowledge ratings.

## Discussion

The first aim of this study was to understand how children talk and learn about machines in museums when visiting with their families. Children generally talked about all aspects of the machine in the learning phase. While they increased their discussions about the whole machine with age, at all ages children were discussing the machine’s parts, such as its wheels, gears, and handles, and, to a lesser extent, its mechanisms. This finding supports the idea that from a young age, children are interested in and motivated to learn not only the facts about an entire artifact but also its less obvious parts and mechanisms (Sobel et al., 2007; Lockhart et al., 2019; Chuey et al., 2020).

However, in the test phase, interesting age effects emerged as older children had greater recall of facts about the whole machine, its parts, and mechanisms and appeared more knowledgeable. This could be due to a combination of factors: First, children from 4 to 8 years make notable gains in understanding how machines work (Leuchter and Naber, 2018; Reuter and Leuchter, 2020), and thus, they would likely know more about all these factors at baseline. Second, older children have better developed memory and other executive functions than younger children (Gathercole, 1998; Ghetti and Angelini, 2008), which may aid in their better recall for all aspects of the machine than younger children. Third, parents and museum staff may have directed children’s learning to these topics more with older children, given that adults likely assume that older children can handle a larger quantity of information and perhaps greater complexity. This possibility is supported by the fact that children’s total, whole, and part talk in the learning phase were related to parent and staff discussions of these respective types of talk, This also supports that some scaffolding may be necessary to draw younger children’s attention to these features and take advantage of the learning opportunities presented to them (Crowley et al., 2001; Fender and Crowley, 2007; Treagust and Duit, 2008; Ferrara et al., 2011; Weisberg et al., 2016). Future work could investigate which aspects of these age-related changes in children’s recall are driven by children or parents and museum staff.

The second aim was to see whether providing a verbal prompt directed to children about mechanisms might affect children’s talk and learning. In general, many children talked about and recalled the facts about the internal parts of the machine, although talk about the machine’s mechanisms occurred less frequently. We found that children that received the component prompt did not discuss parts of the machine or its mechanisms more than participants who received the control prompt during the learning phase or in the test phase. We had hypothesized that focusing children’s attention on the parts of the machine would lead them to discuss its mechanisms more. Future work might explore this relation further by examining how to encourage children to focus on how the components of a machine relate to its internal mechanisms. Because it seems that the minimal verbal prompt did not affect children’s talk, it may have been helpful to scaffold the parents as well so that they could better support their children’s learning. This could have been in the form of a verbal prompt or through the use of cue cards. This museum contains artifacts that may be unfamiliar to 21st century parents, and so, they may have needed additional information or suggestions about the questions to ask staff or the kinds of things they could say to their children to draw their attention to important features.

However, we did find that children who received the component prompt were rated as more knowledgeable than those who received the control prompt by naïve coders. Further, children’s knowledge rating in question 2 was positively correlated with their part and mechanistic utterances in question 1 and question 2. These correlations provide further support for laboratory work showing that discussing internal components and mechanisms in explanations makes one appear more knowledgeable (Lockhart et al., 2019) and that prompting children to explain increases their causal understanding (e.g., Walker et al., 2014).

So why do the subjective knowledge ratings of the children’s explanations differ by condition when the number of part utterances and the number of children generating mechanistic utterances in those explanations did not? We suspect that while the overall number of children making mechanistic utterances about these topics did not differ statistically by condition, the *quality* of their part and mechanistic utterances might differ. As is the case with much of our perception and cognition, examining the sum of children’s explanations may have revealed something more interesting than examining their parts. Based on these findings, children who received a prompt directing their attention to parts and mechanisms may have produced more coherent and logical explanations about those aspects, even if they did not mention them at higher rates.

In general, the effects of the prompts were minimal. What might explain this? First, prior work (e.g., Gelman and Wellman, 1991) suggests that young children understand that the insides of an artifact are important to an artifact’s function and identity. Thus, children in the component prompt condition may not have been as influenced as we had hoped to focus on insides, because they may already be well aware of their importance. However, given that so few children referenced mechanisms in the present dataset, this interpretation is perhaps unlikely. A second possibility is that the prompt was simply too short or subtle or that the control prompt was too well matched to the experimental prompt to reveal differences. That is, both prompts contained the sentence, “go inside and see what you can learn about this machine,” and both prompts referenced the machine “working,” which could have masked differences across conditions. The neutral control prompt was designed to be as equivalent as possible to the component prompt and to direct children’s learning to the machine rather than the store itself. This allowed us to highlight the “inside parts of the machine” specifically in just one prompt to see if that would increase their discussions about mechanisms. On the contrary, a separate potential concern about our prompts was that the control prompt may have directed children’s attention to the historical aspects of the setting. We ruled out this possibility by showing that children in the control prompt condition did not discuss the historical aspect of the setting more than children in the component prompt condition. Future research could investigate whether there are differences in children’s discussions between a component prompt condition vs. a baseline “no prompt” condition. However, pilot data from a previous study conducted by our laboratory in the same setting suggest that a baseline “no prompt” condition may not be a viable option. In that work, we discovered that some small instruction to learn, talk, or ask questions was necessary to get the youngest children to engage in the visit meaningfully. Another option could be to provide a more heavy-handed component prompt, or perhaps a prompt directed at both parents and children, as these findings, compared to previous findings, hint toward the possibility that providing the prompt to both parents and children might be critical to influence engagement in these settings.

This study had a number of limitations; here, we will discuss a few: first, there was a non-significant age difference between the two conditions, where the component prompt condition contained more older children than that in the control prompt condition. This occurred due to random assignment to conditions. When parents inquired about participating, we only asked whether the child fell in the age range of the study, and we alternated condition assignment. In the future, a pseudo-random approach, where children are signed to alternating conditions based on their age in years would reduce age imbalances. However, age was statistically controlled for throughout analyses by entering age in months as a covariate, which alleviates some of this concern. Second, there is a limitation on the generalizability of the current findings given the narrow demographics of our sample (mostly White, highly educated, and high income). Finally, our analyses are also limited to participants’ speech and to assessments of their recall of information. This does not take into account if there were differences in the amount of time children spent exploring the machine or manually interacting with it. It also does not allow for any other measures that might have shown a greater understanding of mechanisms than the ones we used here, such as asking children simple forced-choice questions about what they learned. Parents and museum staff could have also scaffolded children’s learning through gestures and showing children how the machine physically operates. These additional factors could not be examined using the participants’ speech alone.

These findings have implications for visitor experience and exhibit design in historical museums. They confirmed for this specific museum that their exhibits are supporting young children’s learning, including learning about machines and mechanisms, which is well aligned with the local science curricular expectations for grades K-2. This research informed us as well as our partner museum about the potential importance of including some scaffolding or additional information to direct discussions toward mechanisms of machines in their exhibits. Fostering this type of science learning can lead to potential funding opportunities for the museum. For example, we were granted a Partnership Engage Grant from our federal government to examine how children learn in these spaces. This knowledge can open the museum up to exploring funding opportunities for science learning in this space, which is currently a priority area in the funding landscape. At the same time, this is a valuable opportunity for cognitive developmental psychologists, who often conduct work in laboratories to see how learning unfolds in everyday settings and how this aligns with in-lab effects. Notably, we did not find as much (spontaneous) mechanistic talk as we had expected. This finding is in contrast to prior experimental work in the laboratory that suggests that children by early preschool know that internal mechanisms are important to a machine’s operation (e.g., Sobel et al., 2007; Ahl and Keil, 2017; Ahl et al., 2020). This difference demonstrates the value of examining children’s behavior in real-world learning settings.

These findings also show how a simple verbal prompt accompanying an exhibit can influence children’s learning, as it resulted in children producing higher quality explanations of how the machine worked. This finding was particularly valuable for the museum staff as their exhibits are embedded in an outdoor historical village, which cannot take advantage of “traditional exhibit features” that are typically used to enhance learning (e.g., plaques or interactive electronic features). When the museum staff embarks on an explanation about a machine’s functioning in the exhibits, they can begin by drawing children’s attention explicitly to the inside of machines. Afterward, staff could ask children to explain to them how the artifact operates to draw their attention to the mechanistic information about the artifact. This approach could be taken in similar museums, with the use of age-appropriate pamphlets or prompt cards for the parents to use with their children.

## Data Availability Statement

The datasets presented in this study can be found in online repositories. The names of the repository/repositories and accession number(s) can be found at: https://osf.io/dxg7h/.

## Ethics Statement

The studies involving human participants were reviewed and approved by University of Waterloo Research Ethics Committee. Written informed consent to participate in this study was provided by the participants’ legal guardian/next of kin.

## Author Contributions

EA, SN, and SD conceived of the presented idea, planned the experiments, created the coding scheme, and contributed to the data analytic plan. EA ran the participants, acted as a secondary coder, ran the analyses, and wrote the first draft of the manuscript, and all authors contributed to critical feedback, writing, and editing of subsequent drafts. All authors contributed to the article and approved the submitted version.

### Conflict of Interest

The authors declare that the research was conducted in the absence of any commercial or financial relationships that could be construed as a potential conflict of interest.

## Footnotes

^1^Staffs are dressed in 1914 garb and are an integral part of the experience. They are familiar with the historical period and the artifacts. They are generally trained to greet visitors entering an exhibit and to respond to visitors’ questions but to be otherwise unobtrusive. There was a staff member present during each parent–child interaction to preserve the typical experience for visitors (and maintain ecological validity), and thus, the child talk was directed at parents and/or staff.

^2^An independent sample’s *t*-test was conducted to ensure that age was not significantly different between conditions, *t*(52.7) = 1.659, *p* = 0.103.

^3^Thanks to the anonymous reviewer for this suggestion.

^4^This was also examined using all child utterances that were on topic (including utterances such as “yeah” and “mhm.” We found no significant main effect of age (*t* = 1.707, *p* = 0.093), condition (*t* = 0.798, *p* = 0.428), or condition by age interaction (*t* = 0.157, *p* = 0.876). This amount of children’s total talk was also significantly correlated with parent/staff total talk (*r* = 0.462, *p* < 0.001).

^5^We again thank the anonymous reviewer for this suggestion. We created this coding scheme in response to his/her concern.

## References

[ref1] Ahl R. E. Amir D. Keil F. C. (2020). The world within: children are sensitive to internal complexity cues. J. Exp. Child Psychol. 200:104932. 10.1016/j.jecp.2020.104932, PMID: 32783914

[ref2] Ahl R. E. Keil F. C. (2017). Diverse effects, complex causes: children use information about Machines’ functional diversity to infer internal complexity. Child Dev. 88, 828–845. 10.1111/cdev.12613, PMID: 27717127

[ref3] Benjamin N. Haden C. A. Wilkerson E. (2010). Enhancing building, conversation, and learning through caregiver–child interactions in a children’s museum. Dev. Psychol. 46:502. 10.1037/a0017822, PMID: 20210509

[ref4] Callanan M. A. Castañeda C. L. Luce M. R. Martin J. L. (2017). Family science talk in museums: predicting children’s engagement from variations in talk and activity. Child Dev. 88, 1492–1504. 10.1111/cdev.12886, PMID: 28657198

[ref5] Callanan M. A. Legare C. H. Sobel D. M. Jaeger G. J. Letourneau S. McHugh S. R.. (2020). Exploration, explanation, and parent–child interaction in museums. Monogr. Soc. Res. Child Dev. 85, 7–137. 10.1111/mono.12412, PMID: 32175600 PMC10676013

[ref6] Casler K. Kelemen D. (2005). Young children’s rapid learning about artifacts. Dev. Sci. 8, 472–480. 10.1111/j.1467-7687.2005.00438.x, PMID: 16246238

[ref7] Casler K. Kelemen D. (2007). Reasoning about artifacts at 24 months: the developing teleo functional stance. Cognition 103, 120–130. 10.1016/j.cognition.2006.02.006, PMID: 16581053

[ref8] Casler K. Terziyan T. Greene K. (2009). Toddlers view artifact function normatively. Cogn. Dev. 24, 240–247. 10.1016/j.cogdev.2009.03.005

[ref9] Chandler-Campbell I. L. Leech K. A. Corriveau K. H. (2020). Investigating science together: inquiry-based training promotes scientific conversations in parent-child interactions. Front. Psychol. 11:1934. 10.3389/fpsyg.2020.01934, PMID: 32849136 PMC7419620

[ref10] Chuey A. Lockhart K. Sheskin M. Keil F. (2020). Children and adults selectively generalize mechanistic knowledge. Cognition 199:104231. 10.1016/j.cognition.2020.104231, PMID: 32092550

[ref11] Crowley K. Callanan M. A. Jipson J. L. Galco J. Topping K. Shrager J. (2001). Shared scientific thinking in everyday parent-child activity. Sci. Educ. 85, 712–732. 10.1002/sce.1035

[ref12] Erb C. D. Buchanan D. W. Sobel D. M. (2013). Children’s developing understanding of the relation between variable causal efficacy and mechanistic complexity. Cognition 129, 494–500. 10.1016/j.cognition.2013.08.002, PMID: 24041835

[ref13] Fender J. G. Crowley K. (2007). How parent explanation changes what children learn from everyday scientific thinking. J. Appl. Dev. Psychol. 28, 189–210. 10.1016/j.appdev.2007.02.007

[ref14] Ferrara K. Hirsh-Pasek K. Newcombe N. S. Golinkoff R. M. Lam W. S. (2011). Block talk: spatial language during block play. Mind Brain Educ. 5, 143–151. 10.1111/j.1751-228X.2011.01122.x

[ref45] Frazier B. N. Gelman S. A. Wellman H. M. (2009). Preschoolers’ search for explanatory information within adult–child conversation. Child Dev. 80, 1592–1611. 10.1111/j.1467-8624.2009.01356.x 19930340 PMC2784636

[ref15] Gathercole S. E. (1998). The development of memory. J. Child Psychol. Psychiatry 39, 3–27. 10.1017/S0021963097001753, PMID: 9534084

[ref16] Gelman S. A. Wellman H. M. (1991). Insides and essences: early understandings of the non-obvious. Cognition 38, 213–244. 10.1016/0010-0277(91)90007-Q, PMID: 2060270

[ref17] German T. P. Johnson S. C. (2002). Function and the origins of the design stance. J. Cogn. Dev. 3, 279–300. 10.1207/S15327647JCD0303_2

[ref18] Ghetti S. Angelini L. (2008). The development of recollection and familiarity in childhood and adolescence: evidence from the dual-process signal detection model. Child Dev. 79, 339–358. 10.1111/j.1467-8624.2007.01129.x, PMID: 18366427

[ref19] Greif M. L. Kemler Nelson D. G. Keil F. C. Gutierrez F. (2006). What do children want to know about animals and artifacts? Domain-specific requests for information. Psychol. Sci. 17, 455–459. 10.1111/j.1467-9280.2006.01727.x, PMID: 16771792 PMC3034738

[ref20] Haden C. A. Jant E. A. Hoffman P. C. Marcus M. Geddes J. R. Gaskins S. (2014). Supporting family conversations and children’s STEM learning in a children’s museum. Early Child. Res. Q. 29, 333–344. 10.1016/j.ecresq.2014.04.004

[ref21] Jant E. A. Haden C. A. Uttal D. H. Babcock E. (2014). Conversation and object manipulation influence children’s learning in a museum. Child Dev. 85, 2029–2045. 10.1111/cdev.12252, PMID: 24773335

[ref22] Kelemen D. (1999). The scope of teleological thinking in preschool children. Cognition 70, 241–272. 10.1016/S0010-0277(99)00010-4, PMID: 10384737

[ref23] Kemler Nelson D. G. Holt M. B. Egan L. C. (2004). Two-and three-year-olds infer and reason about design intentions in order to categorize broken objects. Dev. Sci. 7, 543–549. 10.1111/j.1467-7687.2004.00378.x, PMID: 15603287

[ref24] Kominsky J. F. Zamm A. P. Keil F. C. (2018). Knowing when help is needed: a developing sense of causal complexity. Cogn. Sci. 42, 491–523. 10.1111/cogs.12509, PMID: 28675496 PMC5754261

[ref25] Landis J. R. Koch G. G. (1977). The measurement of observer agreement for categorical data. Biometrics 33, 159–174. 10.2307/2529310, PMID: 843571

[ref26] Legare C. H. Gelman S. A. Wellman H. M. (2010). Inconsistency with prior knowledge triggers children’s causal explanatory reasoning. Child Dev. 81, 929–944. 10.1111/j.1467-8624.2010.01443.x, PMID: 20573114 PMC3039682

[ref27] Leuchter M. Naber B. (2018). Studying children’s knowledge base of one-sided levers as force amplifiers. J. Res. Sci. Teach. 56, 91–112. 10.1002/tea.21470

[ref28] Lockhart K. L. Chuey A. Kerr S. Keil F. C. (2019). The privileged status of knowing mechanistic information: an early epistemic bias. Child Dev. 90, 1772–1788. 10.1111/cdev.13246, PMID: 31106424

[ref29] Matan A. Carey S. (2001). Developmental changes within the core of artifact concepts. Cognition 78, 1–26. 10.1016/S0010-0277(00)00094-9, PMID: 11062320

[ref30] Medina C. Sobel D. M. (2020). Caregiver–child interaction influences causal learning and engagement during structured play. J. Exp. Child Psychol. 189:104678. 10.1016/j.jecp.2019.104678, PMID: 31635828

[ref31] Michigan Department of Education (2015). Michigan K-12 Standards [Science]. Available at: https://www.michigan.gov/documents/mde/K12_Science_Performance_Expectations_v5_496901_7.pdf (Accessed April, 2018).

[ref32] Ontario Ministry of Education (2007). The Ontario curriculum grades 1–8: Science and Technology [Program of Studies]. Available at: http://www.edu.gov.on.ca/eng/curriculum/elementary/scientec18currb.pdf (Accessed April, 2018).

[ref33] Pagano L. C. Haden C. A. Uttal D. H. (2020). Museum program design supports parent–child engineering talk during tinkering and reminiscing. J. Exp. Child Psychol. 200:104944. 10.1016/j.jecp.2020.104944, PMID: 32791381

[ref34] Reuter T. Leuchter M. (2020). Children’s concepts of gears and their promotion through play. J. Res. Sci. Teach. 58, 69–94. 10.1002/tea.21647

[ref35] RoweM. L.LeechK. A.CabreraN. (2017). Going beyond input quantity: Wh-questions matter for toddlers’ language and cognitive development. Cogn. Sci. 41, 162–179. 10.1111/cogs.12349, PMID: 26923546

[ref36] Sobel D. M. Jipson J. L. (2016). Cognitive Development in Museum Settings: Relating Research and Practice. New York, NY: Routledge.

[ref37] Sobel D. M. Letourneau S. M. Legare C. H. Callanan M. (2020). Relations between parent-child interaction and children’s engagement and learning at a museum exhibit about electric circuits. Dev. Sci. 24:e13057 10.1111/desc.13057 33108708

[ref38] Sobel D. M. Yoachim C. M. Gopnik A. Meltzoff A. N. Blumenthal E. J. (2007). The blicket within: Preschoolers’ inferences about insides and causes. J. Cogn. Dev. 8, 159–182. 10.1080/15248370701202356, PMID: 18458796 PMC2367333

[ref39] Tenenbaum H. R. Prior J. Dowling C. L. Frost R. E. (2010). Supporting parent–child conversations in a history museum. Br. J. Educ. Psychol. 80, 241–254. 10.1348/000709909X470799, PMID: 19719907

[ref40] Treagust D. F. Duit R. (2008). Conceptual change: a discussion of theoretical, methodological and practical challenges for science education. Cult. Stud. Sci. Educ. 3, 297–328. 10.1007/s11422-008-9090-4

[ref41] Walker C. M. Lombrozo T. Legare C. H. Gopnik A. (2014). Explaining prompts children to privilege inductively rich properties. Cognition 133, 343–357. 10.1016/j.cognition.2014.07.008, PMID: 25128793

[ref42] Weatherhead D. Nancekivell S. (2018). Brungarians use it differently! Children’s understanding of artifact function as a cultural convention. J. Cogn. Cult. 18, 89–103. 10.1163/15685373-12340019

[ref43] Weisberg D. S. Hirsh-Pasek K. Golinkoff R. M. Kittredge A. K. Klahr D. (2016). Guided play: principles and practices. Curr. Dir. Psychol. Sci. 25, 177–182. 10.1177/0963721416645512

[ref44] Willard A. K. Busch J. T. Cullum K. A. Letourneau S. M. Sobel D. M. Callanan M.. (2019). Explain this, explore that: a study of parent–child interaction in a children’s museum. Child Dev. 90, e598–e617. 10.1111/cdev.13232, PMID: 30866040 PMC6850333

